# EPIGENETIC DIFFERENCE BETWEEN INSOMNIA GROUP AND NONINSOMNIA GROUP IN PARTICIPANTS WITH KNEE PAIN

**DOI:** 10.1093/geroni/igad104.2767

**Published:** 2023-12-21

**Authors:** Soamy Montesino Goicolea, Lingsong Meng, Zhiguang Huo, Thomas C Foster, Burel R Goodin, Joseph L Riley III, Roger Fillingim, Yenisel Cruz-Almeida

**Affiliations:** University of Florida, Gainesville, Florida, United States; The University of Florida, Gainesville, Florida, United States; The University of Florida, Gainesville, Florida, United States; University of Florida, Gainesville, Florida, United States; Washington University, St. Louis, Missouri, United States; University of Florida, Gainesville, Florida, United States; The University of Florida, Gainesville, Florida, United States; The University of Florida, Gainesville, Florida, United States

## Abstract

Chronic knee pain is a common condition that often co-occurs with insomnia. Epigenetic modifications, such as DNA methylation, may play a role in the sleep-pain relationship. In this study, we aimed to identify differentially methylated CpGs/regions and enriched genomic pathways associated with insomnia in participants with knee pain (KP). We recruited 140 cognitively healthy middle to older-aged adults with self-reported KP and extracted DNA from peripheral blood. MethylationEPIC arrays were used for methylation data quantification, R package minfi was used for data preprocessing and quality control, and Ingenuity Pathway Analysis (IPA) was used to explore functional implications of differential methylation in pathways related to insomnia. Annotated genes within ±5kb of the putative differentially methylated regions (DMRs, p< 0.05) were subjected to the IPA analysis. There were no significant differences in sex or race between the two groups (p>0.05). We observed an over-representation of younger participants in the insomnia group (p=0.005) and also from the University of Florida (p=0.008). At raw p < 0.05 cutoff, we identified a total of 20,006 CpG probes, including 12,674 hypermethylated, and 7,332 hypomethylated CpG probes. Pathway analysis using IPA identified multiple immune-related pathways enriched with insomnia-related differentially methylated regions (DMRs), including the antigen presentation pathway (p=3.39E-08). Verapamil was identified as the most significant upstream regulator (p=8.8E-07). Our findings suggest the importance of epigenetic regulation of the immune system in the sleep-pain relationship and highlight the need for further research to understand the epigenetic contributions to sleep disorders in individuals with chronic pain.

